# Potential generation of geographical inequities by the introduction of primary percutaneous coronary intervention for the management of ST segment elevation myocardial infarction

**DOI:** 10.1186/1476-072X-6-43

**Published:** 2007-09-23

**Authors:** Augustine Pereira, Aphrodite Niggebrugge, John Powles, David Kanka, Georgios Lyratzopoulos

**Affiliations:** 1 NHS East of England, Cambridge, UK; 2 Eastern Region Public Health Observatory, Cambridge, UK; 3 Institute of Public Health, Department of Public Health and Primary Care, University of Cambridge, Cambridge, UK; 4 Suffolk Primary Care Trust, Suffolk, UK

## Abstract

**Background:**

Primary Percutaneous Coronary Intervention (PCI) is more efficacious than thrombolysis in the management of acute myocardial infarction, but, because of the requirement for prompt treatment, there are practical challenges in developing such services. We examined the proportion of patients with ST segment Elevation Myocardial Infarction (STEMI) who could receive timely treatment from a primary Percutaneous Coronary Intervention (PCI) service assuming different geographical locations of potential treatment centres in three English counties.

**Methods and results:**

Information on the residential location of patients with new STEMI hospitalisations recorded in Hospital Episodes Statistics was analysed and the proportion of episodes of STEMI within 60' and 45' travel time isochrones from potential primary PCI centres in three English counties was calculated. There were on average 1,815 new STEMI hospitalisations per year occurring in the studied population. Introduction of a primary PCI service in one, two or three potential treatment centres would have covered respectively 28%, 73% and 90% of such episodes within 60 minutes travel time, and 17%, 51% and 69% within 45 minutes travel time.

**Conclusion:**

In the study context, a primary PCI service in an existing tertiary centre would only cover a minority of STEMI events and would generate geographical inequities. A two-centre model would improve coverage and equity considerably, but may be associated with practical, clinical quality and financial challenges.

## Background

Although primary Percutaneous Coronary Intervention (PCI) is more efficacious for the treatment of ST-segment Elevation Myocardial Infarction (STEMI) compared with thrombolysis, [1-6] the comparative advantage is highly time dependent. The European Society of Cardiology (ESC) currently recommends a limit of 90 minutes from first medical contact to initiation of treatment [7,8] This means that travel time considerations are of paramount importance in accessing such a service, particularly so in rural areas. The United Kingdom's Department of Health has recently published a policy document, supporting the use of primary PCI for the management of STEMI [9] Norfolk, Suffolk and Cambridgeshire are three predominantly rural English counties, with a mid-2004 resident population estimate of 2.24 million, living in an area of approximately 4,850 square miles. At the time of the study, management of STEMI with primary PCI was not routinely used in the three counties. Given the spatial distribution of STEMIs relative to potential treatment centres, we set out to determine the incremental coverage of one, two and three centres for a potential future primary PCI service.

## Methods

### Travel time cut-offs

It was assumed that in order to administer the treatment within existing guideline times of 90 minutes, [7,8,10,11] there is a maximum period of 60 minutes available to transport a patient from the place of STEMI onset to a primary PCI centre. This assumes that the time required for transporting the patient in and out of the ambulance, and from arrival at the hospital door to first balloon inflation (door-to-balloon time) is less than 30 minutes. In supplementary analysis, an additional period of 15 minutes was allowed for transporting the patient in and out of the ambulance, and for door-to-balloon time, therefore reducing the time available for travel from the place of onset to the hospital door to a maximum of 45 minutes.

### Geographical analysis

New STEMI-related hospitalisations [defined as Hospital Episode Statistics (HES) records with an ICD-10 primary diagnosis code of I210, I211, I212, I220, I221 or I228, and no record of a previous STEMI hospitalisation within the 28 days prior to admission] in patients aged 18 and over were analysed for the five-year period 1998–99 to 2002–03. Hospital Episodes Statistics do not record the location of STEMI onset (and ambulance pick-up), and obtaining such information was not possible within the resources available. Therefore, the patient's post-code of residence was used as a surrogate marker of STEMI onset location. This assumes that STEMI onset location outside the patients' own home was, on average, randomly scattered around their residential post-code, both in terms of distance and travel time to potential treatment centres. Subsequently, all new STEMI episodes recorded in HES were mapped to the respective patient postcodes using Geographical Information System (GIS) software (ArcGIS version 9). The shortest route to potential primary PCI centres was identified and the weighted average mean travel time to each potential primary PCI centre was calculated assuming 'blue light' ambulance travel speeds through the routes identified.

### Choice of one-, two- and three-centre potential locations

The potential primary PCI centres examined in this study represent the location of an existing tertiary cardiac centre (one-centre scenario), or the location of an existing teaching hospital in addition to the cardiac centre (two-centre scenario). The third location, in the three-centre scenario, relates to an existing District General Hospital. It was chosen from a total of another six hospitals of a similar type, compared with the two-centre scenario, as it provided the relatively greatest gain in coverage

### Coverage estimates

STEMI events relating to patients whose residence was within the travel time limits to a potential primary PCI centre were assumed to have had access to primary PCI. The incremental coverage for one-centre, two centre and three-centre scenarios were calculated using SPSS version 11.5.

## Results

During the five-year study period, there were on average 1,815 new STEMI hospitalisations per year in Norfolk Suffolk and Cambridgeshire residents in National Health Service (NHS) hospitals within these three counties, recorded in the HES dataset.

**Figure 1:**
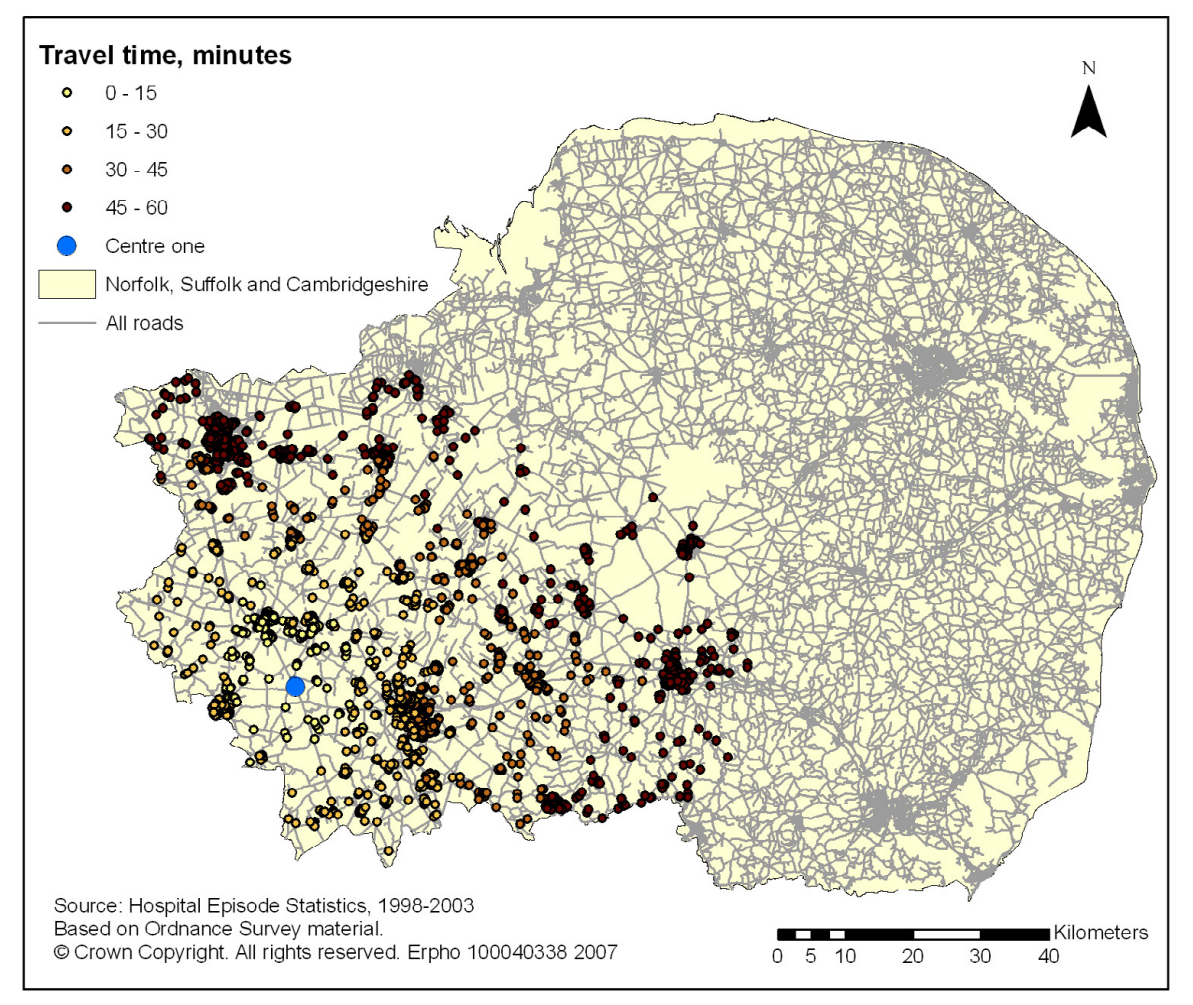
GIS mapping of all new STEMI patients (1998–99 to 2002–03) across Norfolk Suffolk and Cambridgeshire within 60 minutes isochrones of travel times (blue light ambulance) from centre one.

**Figure 2:**
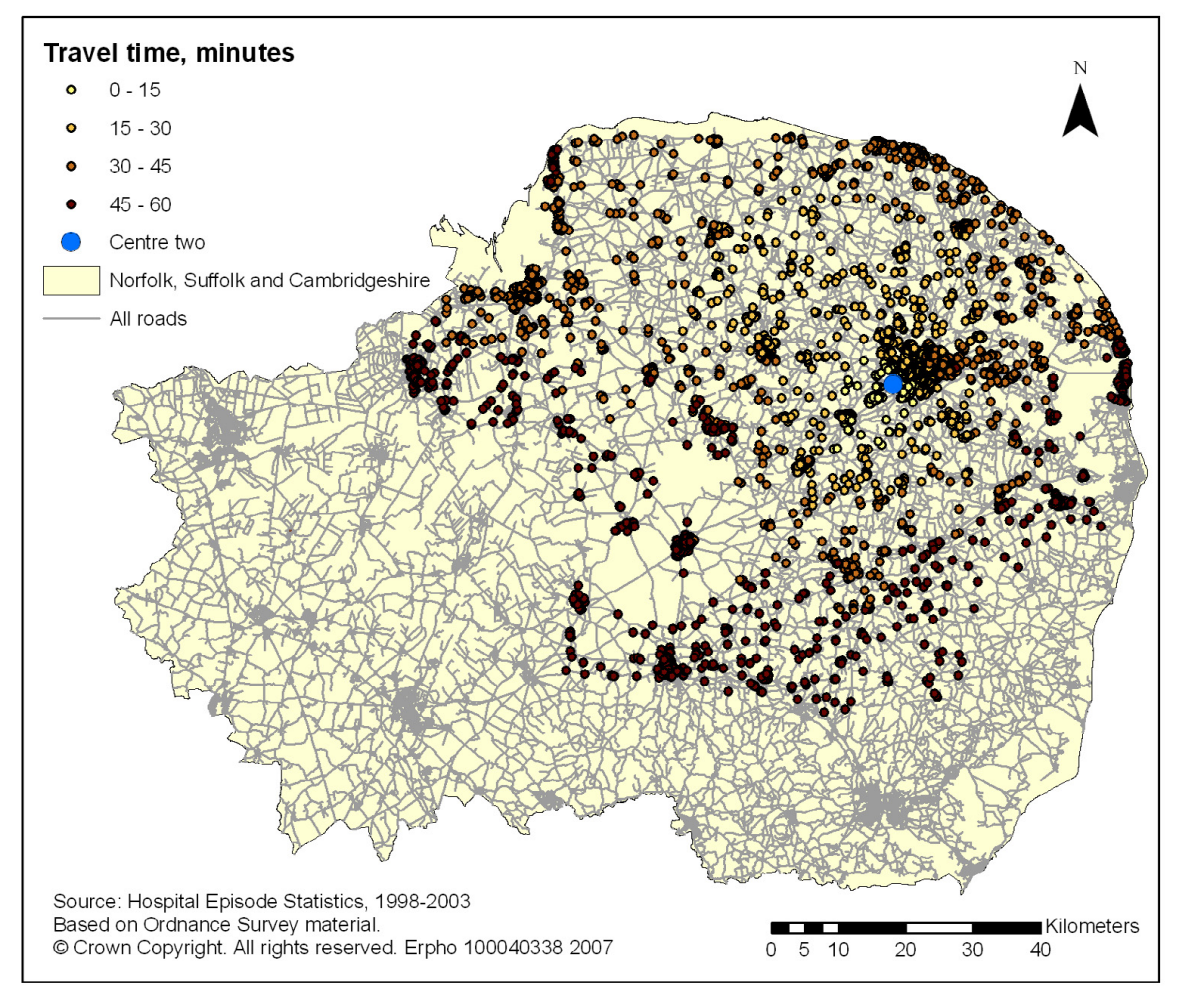
GIS mapping of all new STEMI patients (1998–99 to 2002–03) across Norfolk Suffolk and Cambridgeshire within 60 minutes isochrones of travel times (blue light ambulance) from centre two.

**Figure 3:**
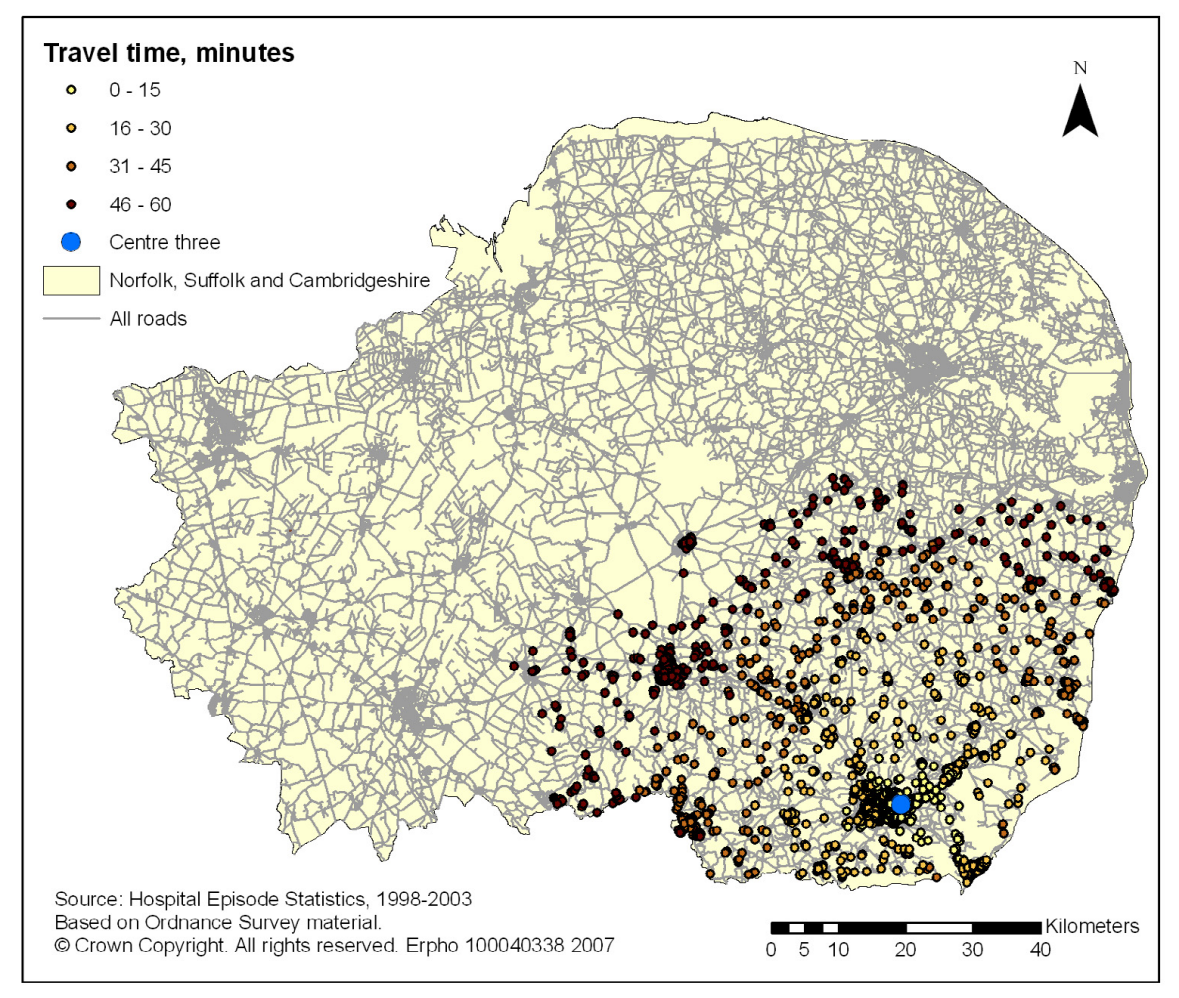
GIS mapping of all new STEMI patients (1998–99 to 2002–03) across Norfolk Suffolk and Cambridgeshire within 60 minutes isochrones of travel times (blue light ambulance) from centre three.

The coverage of all new STEMI hospitalisation for one, two and three potential primary PCI centres is described in table 1. The coverage increased from 28% and 17% for an one-centre model alone to 73% and 51% for the two-centre and 90% and 69% for the three-centre models, using the 60 and 45 minute travel time limits respectively (see figures 1, 2 and 3).

**Table 1:** Coverage of STEMI events by the provision of primary PCI using one, two or three-centre models.

Model of provision	Coverage at 60 minutes, % (95% CI)	Coverage at 45 minutes, % (95% CI)
One centre	28.4 (27.5–29.3)	16.5 (15.8–17.2)
Two centre	73.4 (72.5–74.3)	51.3 (50.3–52.3)
Three centre	89.8 (89.2–90.4)	69.0 (68.1–69.9)

## Discussion

In the study setting and using the European Society of Cardiology Guidelines about target time for administration of primary PCI, introduction of a PCI service for the management of STEMI in a single centre (one-centre location) will be associated with substantial geographical inequity. A two-centre provision greatly improves geographical coverage and a three-centre option raises coverage further. However, the latter two scenarios may be associated with very important resource implications, staffing and clinical quality challenges.

This study used an innovative methodology to estimate the proportion of STEMI events that can benefit from a primary PCI service in three counties of a European country. It used five years of event data and estimated proportional coverage for potential primary PCI centres taking into account shortest travel route to a potential centre, assuming blue light ambulance travel speeds. Using geographical analysis to estimate proportional coverage for a primary PCI service development can usefully underpin and facilitate the planning, development and monitoring of such healthcare services.

Although the application of the methodology relates to one distinct geographical area, it can easily be extended to other areas, and indeed also applied to different healthcare services if mean travel time considerations are of relevance to policy-making.

Our study used actual event data, over a five-year period. We believe that this is superior to calculations based on general population density alone – as the latter may not correlate highly with coronary heart disease burden, due to the uneven spatial distribution of demographic parameters, such as age and social deprivation. Clearly, a proportion of patients developing a STEMI event will not be eligible for primary PCI, on account of medical reasons. However, this consideration is independent of the geographical location, and the percentage coverage estimates presented here apply to those patients eligible for primary PCI.

There are certain limitations of our study. Firstly, we used the post-code of residence of patients who suffered from a STEMI event that resulted in hospitalisation as a surrogate marker for event location. The onset of some STEMI events will take place outside the patients' residence, e.g. in the community. However, overall, this is unlikely to have introduced differential bias in relation to travel time to a potential PCI centre. We assumed that the distribution of travel times from the home addresses of patients admitted with STEMIs provided an unbiased estimate of the distribution of travel times from the actual site of onset – in other words that the instances when the actual site of onset was further from the treatment centre of interest would be approximately offset by the instances when it was closer.

Secondly, we have treated the three counties of Norfolk, Suffolk & Cambridgeshire as an 'island'. However, the tertiary centre relating to the one-centre option provides cardiothoracic services not only to the three counties examined in this study, but also to at least another four neighbouring counties. Our results therefore underestimate the absolute population that could be served if such a service was introduced. Nevertheless, this option will certainly introduce significant inequity in care provided to STEMI patients within the population of primary interest to this study. As the number of tertiary cardiac centres is unevenly distributed between English counties, future work should address the question of optimal service configuration for a potential primary PCI service development at inter-regional and national level.

## Conclusion

A primary PCI service in one existing tertiary centre would only cover a minority of STEMI events and would generate geographical inequities. A two-centre option improves access greatly. However, setting up a primary PCI service in a centre where one doesn't exists requires significant capital expenditure to expand facilities and recruit, train and employ relevant staff. In addition, there may be challenges in maintaining clinical quality in hospitals with a relative low throughput of patients. If newer evidence would indicate that certain PCI treatment protocols may be superior to thrombolysis beyond the time period from symptom onset currently recommended, [12] assumptions about time periods modelled should be likewise informed in subsequent analyses.

## Abbreviations

PCI – Percutaneous Coronary Intervention

STEMI – ST-segment Elevation Myocardial Infarction

HES – Hospital Episode Statistics

GIS – Geographical Information System

## Competing interests

The author(s) declare that they have no competing interests.

## Authors' contributions

The study idea was originally conceived by GL, and was developed further with contribution from AP, JP, AN and DK. AP wrote the first draft, analysed data, and coordinated all revisions. AN under-took the geographical analysis. All authors commented on drafts of the paper, and helped interpret the findings and identify policy implications. All authors have read and approved the final manuscript. AP is guarantor.

## References

[B1] Keeley EC, Boura JA, Grines CL (2003). Primary angioplasty versus intravenous thrombolytic therapy for acute myocardial infarction: a quantitative review of 23 randomised trials. Lancet.

[B2] Weaver WD, Cerqueira M, Hallstrom AP, Litwin PE, Martin JS, Kudenchuk PJ, Eisenberg M (1993). Prehospital-initiated vs hospital-initiated thrombolytic therapy. The Myocardial Infarction Triage and Intervention Trial. JAMA.

[B3] Zijlstra F (2003). Angioplasty vs thrombolysis for acute myocardial infarction: a quantitative overview of the effects of interhospital transportation. Eur Heart J.

[B4] Michels KB, Yusuf S (1995). Does PTCA in acute myocardial infarction affect mortality and reinfarction rates? A quantitative overview (meta-analysis) of the randomized clinical trials. Circulation.

[B5] Zijlstra F, Patel A, Jones M, Grines CL, Ellis S, Garcia E, Grinfeld L, Gibbons RJ, Ribeiro EE, Ribichini F, Granger C, Akhras F, Weaver WD, Simes RJ (2002). Clinical characteristics and outcome of patients with early (<2 h), intermediate (2-4 h) and late (>4 h) presentation treated by primary coronary angioplasty or thrombolytic therapy for acute myocardial infarction. Eur Heart J.

[B6] Weaver WD, Simes RJ, Betriu A, Grines CL, Zijlstra F, Garcia E, Grinfeld L, Gibbons RJ, Ribeiro EE, DeWood MA, Ribichini F (1997). Comparison of primary coronary angioplasty and intravenous thrombolytic therapy for acute myocardial infarction: a quantitative review. JAMA.

[B7] Silber S, Albertsson P, Aviles FF, Camici PG, Colombo A, Hamm C, Jorgensen E, Marco J, Nordrehaug JE, Ruzyllo W, Urban P, Stone GW, Wijns W (2005). Guidelines for percutaneous coronary interventions. The Task Force for Percutaneous Coronary Interventions of the European Society of Cardiology. Eur Heart J.

[B8] Bassand JP, Danchin N, Filippatos G, Gitt A, Hamm C, Silber S, Tubaro M, Weidinger F (2005). Implementation of reperfusion therapy in acute myocardial infarction. A policy statement from the European Society of Cardiology. Eur Heart J.

[B9] Boyle R (2006). Mending hearts and brains, Clinical case for change..

[B10] Caputo RP, Ho KK, Stoler RC, Sukin CA, Lopez JJ, Cohen DJ, Kuntz RE, Berman A, Carrozza JP, Baim DS (1997). Effect of continuous quality improvement analysis on the delivery of primary percutaneous transluminal coronary angioplasty for acute myocardial infarction. Am J Cardiol.

[B11] Moscucci M, Eagle KA (2006). Door-to-balloon time in primary percutaneous coronary intervention: is the 90-minute gold standard an unreachable chimera?. Circulation.

[B12] Armstrong PW (2006). A comparison of pharmacologic therapy with/without timely coronary intervention vs. primary percutaneous intervention early after ST-elevation myocardial infarction: the WEST (Which Early ST-elevation myocardial infarction Therapy) study. Eur Heart J.

